# Radiation-Stimulated Formation of Two-Dimensional Structures Based on Calcium Silicide

**DOI:** 10.3390/nano12203623

**Published:** 2022-10-16

**Authors:** Aigul F. Zinovieva, Vladimir A. Zinovyev, Natalia P. Stepina, Vladimir A. Volodin, Aleksey Y. Krupin, Aleksey V. Kacyuba, Anatoly V. Dvurechenskii

**Affiliations:** 1 Rzhanov Institute of Semiconductor Physics, Siberian Branch of Russian Academy of Sciences, 630090 Novosibirsk, Russia; 2 Department of Physics, Novosibirsk State University, 630090 Novosibirsk, Russia; 3 Depertment of Physics, Novosibirsk State Technical University, 630073 Novosibirsk, Russia

**Keywords:** calcium silicide, two-dimensional layer, Raman spectroscopy, electron irradiation

## Abstract

The formation of CaSi_2_ polycrystalline structures under the postgrowth electron irradiation of epitaxial CaF_2_/Si(111) films with embedded thin Si layers was studied. The dependence on the electron exposure time was investigated for two types of structures with different film thicknesses. The optimal conditions for the formation of two-dimensional CaSi_2_ structures were found. Raman spectra of the structures after a 1 min electron irradiation demonstrated only one pronounced peak corresponding to the vibrations of Si atoms in the plane of the calcium-intercalated two-dimensional Si layer. An increase in the exposure time resulted in the transition from two- to three-dimensional CaSi_2_ structures having more complex Raman spectra with additional peaks typical of bulk CaSi_2_ crystals. Based on the results of microscopic studies and transport measurements, a model explaining the observed effects was proposed.

## 1. Introduction

In recent decades, there has been increased interest in the synthesis of graphene-like structures based on silicon. Many works have been devoted to the problem of silicene production and its device applications (see reviews [1,2] and references therein). However, obtaining a two-dimensional material that contains a single silicon layer remains an unsolved problem. The monolayer silicene is expected to have unique properties, such as tunable band gaps [3], a quantum spin Hall effect [4], high-temperature superconductivity [5] and giant magnetoresistance [6]. Despite great progress having been achieved in the epitaxial synthesis of silicene [7,8], its poor air stability makes its device application difficult [9]. Recently, Yaokawa et al. [10] reported the formation of bilayer silicene (BLSi) by treating calcium-intercalated monolayer silicene (CaSi_2_) with a BF_4_-based ionic liquid. The bilayer silicenes were sandwiched between planar crystals of CaF_2_ and/or CaSi_2_. The authors [10] predicted that BLSi would be more stable in air than monolayer silicene, because it has a low density of dangling bonds. We believe that the CaF_2_–CaSi_2_ system is a very prospective basis for future device applications; thus, we focus on CaSi_2_, an attractive precursor of BLSi. Recently, it was found that calcium-intercalated silicon atomic layers in CaSi_2_ exhibit electronic properties typical of graphene-like materials [11]. Many scientific groups are currently involved in the research of calcium silicides [12,13,14,15,16,17,18,19,20,21]. A variety of methods has been proposed to produce calcium silicides with different compositions that exhibit semimetallic or semiconducting properties. However, the two-dimensional structures based on calcium silicides have not been obtained. There have been attempts to grow two-dimensional silicon layers on CaF_2_ using the molecular beam epitaxy method, but up to now, there has been no significant success in this direction. As a rule, in the case of conventional silicon deposition on CaF_2_, three-dimensional nuclei are formed due to a poor wettability of the silicon on this fluoride [22,23], preventing a two-dimensional layer growth.

Earlier, we proposed a method for CaSi_2_ synthesis using electron beam irradiation during the growth of CaF_2_ layers with molecular beam epitaxy (MBE) [19,24,25]. It was assumed that the formation of CaSi_2_ occurs through the stimulated decomposition of CaF_2_ into Ca and F [26]. Fluorine is desorbed from the surface, and remaining calcium atoms bind chemically with silicon atoms, which come from the Si substrate at sufficiently high temperatures (>300 °C) under electron irradiation [24]. Calcium silicide produced in this way is a nonhomogeneous three-dimensional material representing a triangular network of elongated crystallites protruding from the surface of the CaF_2_ film by tens of nanometers. These crystallites are oriented along directions {1–10} and have a characteristic length of ~1 μm. We recently found a way to produce more homogeneous CaSi_2_ films [27]. The idea is to introduce additional intermediate silicon layers into the growing CaF_2_ film. In this case, the CaSi_2_ film growth under simultaneous e-beam irradiation occurs in a layer-by-layer mode. Another opportunity to increase the CaSi_2_ film homogeneity is postgrowth electron irradiation after CaF_2_ deposition [25].

In the present paper, we used both of these approaches to obtain a two-dimensional material based on calcium silicide. The electron beam was used to irradiate the surface of an already grown CaF_2_ film with embedded thin Si layers. By adjusting the time of electronic exposure, the CaF_2_ film thickness and the temperature of the substrate, it was possible to tune the properties of the material synthesized under an electron beam and obtain two-dimensional CaSi_2_ regions.

## 2. Materials and Methods

The experiments were conducted in the “Katun-100” MBE unit equipped with a CaF_2_ effusion source with a graphite crucible under ultrahigh vacuum conditions. The structures were synthesized on Si (111) substrates with a 100 mm diameter. Before the growth, the silicon substrates underwent a double surface cleaning. After a standard chemical treatment, the protective silicon oxide layer was formed. This protective layer was removed in the MBE chamber at 720 °C in a weak Si flux until the appearance of a 7 × 7 superstructure fixed with reflection high-energy electron diffraction (RHEED), after which a 50 nm thick buffer Si layer was grown at a temperature of 550 °C.

**Figure 1:**
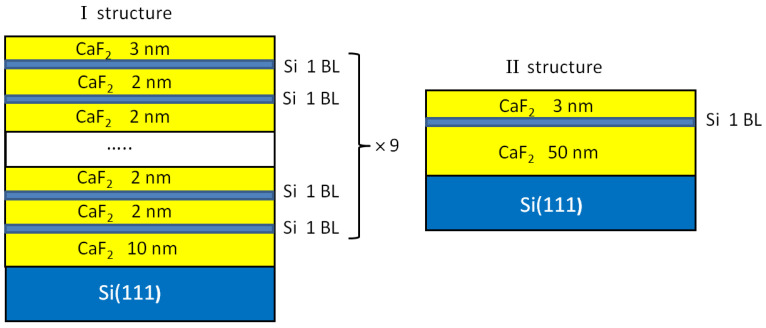
A schematic representation of two structures under study.

The epitaxial CaF_2_ film growth was carried out at a deposition rate of ~2 nm/min at a substrate temperature of 550 °C. During the growth, the selected area of the CaF_2_ film was controlled with RHEED using the following electron beam parameters: an acceleration voltage of 20 keV and a current density of 50 μA/m^2^. The beam incidence angle was 4°. Two structures of different CaF_2_ film thicknesses (29 nm and 53 nm) with incorporated Si layers were grown at a temperature of 550 °C (Figure 1). The first structure contained 9 Si layers with a thickness of 0.3 nm (~1 BL of silicon), separated by 2 nm thick CaF_2_ interlayers. Si layers were grown on a 10 nm thick CaF_2_ film. The last Si layer was covered with a 3 nm thick CaF_2_ layer. The rate of Si deposition was ~0.6 nm/min. The second structure contained 1 BL Si layer grown on a 50 nm thick CaF_2_ film and covered with a 3 nm CaF_2_ layer. Just after the growth, the already-formed films were exposed to electron irradiation produced with an electron beam used for RHEED under ultrahigh vacuum conditions. The electron exposure times were 1, 2, 4 and 10 min, and each time, the electron beam was moved to a new place on the substrate. The substrate temperature during electron irradiation was kept at 550 °C. As a result, the electron-beam-modified areas were formed on the surface of the grown film. These areas were strips with a metallic luster with a length of 3–4 cm and a width of 2 mm. One of the strips was obtained during the control of the film growth with RHEED, and was used later for testing the Raman measurements. The strips were investigated with energy-dispersive X-ray spectroscopy (EDX), atomic force microscopy (AFM), scanning electron microscopy (SEM) and Raman spectroscopy. The thickness of the grown films was controlled with ellipsometry [28]. The conductivity and magnetoresistance were measured on the strips as functions of the electron irradiation time. Contacts for transport measurements were created through the soldering of silver wires using indium solder. The magnetoresistance and temperature dependences of the conductivity were measured using an SR850 synchronous amplifier in a transport helium Dewar vessel in a magnetic field up to 4 T.

## 3. Results and Discussion

**Figure 2:**
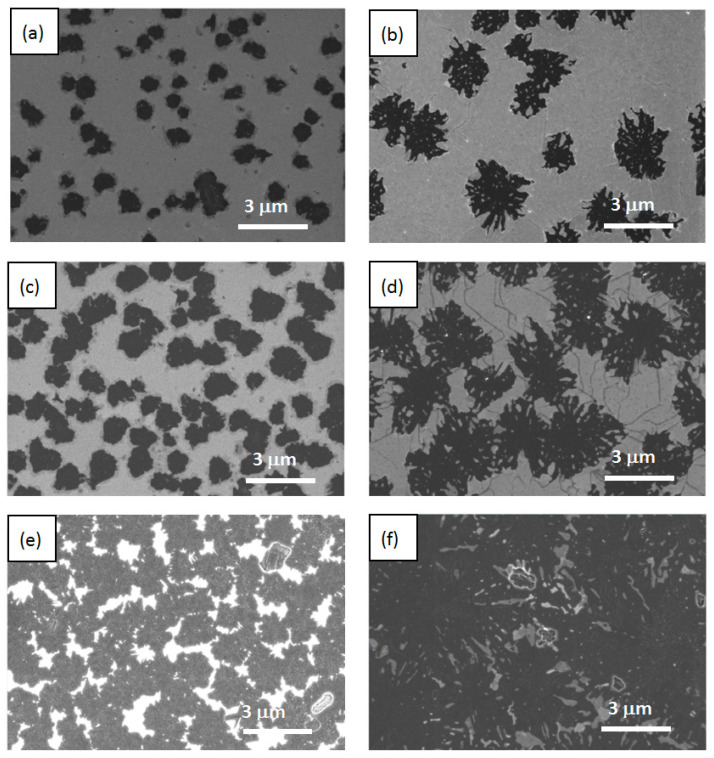
SEM data obtained for structures with different thicknesses. Left column—29 nm thick film; right column—53 nm thick film. Panels (**a**,**b**) correspond to a 2 min exposure time. Panels (**c**,**d**) correspond to a 4 min exposure time. Panels (**e**,**f**) correspond to a 10 min exposure time.

**Figure 3:**
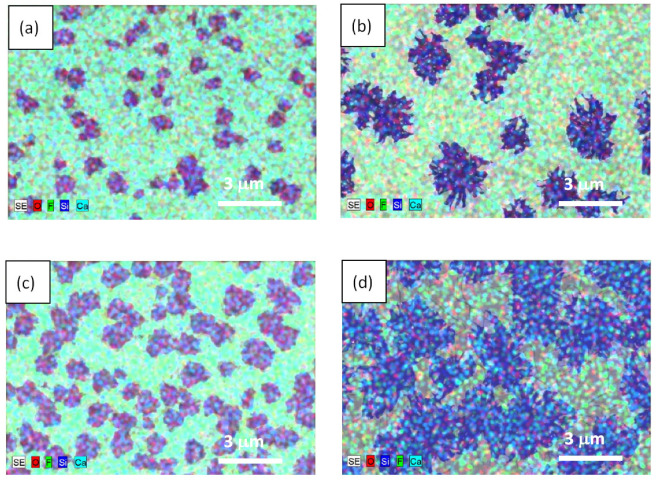
(**a**)—Energy-dispersive X-ray spectroscopy data obtained for the structures with different thicknesses of epitaxial films. Left column—29 nm thick film; right column—53 nm thick film. Panels (**a**,**b**) correspond to a 2 min exposure time. Panels (**c**,**d**) (correspond to a 4 min exposure time.

As a result of studies with SEM (Figure 2) and EDX (Figure 3), it was found that at short electron beam exposure times (1–2 min) on the CaF_2_ surface, individual microstructures were formed. The size and shape of the obtained structures depended on the thickness of the epitaxial CaF_2_ films. According to RHEED measurements, these structures were polycrystalline. The RHEED images obtained in analogous experimental conditions were presented in [25].

In the case of a 29 nm thick film, the small structures resembling rounded spots with a characteristic size of ~1 μm formed on the surface (see left panels in Figure 2 and Figure 3). For a 53 nm thick film, larger snowflake-like structures formed several times (see right panels in Figure 2 and Figure 3). The density of the polycrystalline structures on the surface of a 29 nm thick film was a few times higher than that for a 53 nm thick film. In both cases with increasing irradiation times (see from top to bottom panels in Figure 2 and Figure 3), the area occupied by these structures increased. At a 10 min exposure time, for a 53 nm thick film, they overlapped, forming almost a continuous layer (Figure 2f). Combining the results of the EDX and Raman studies (see below), one could conclude that the resulting polycrystalline structures contained Ca and Si.

An analysis of the SEM data revealed another interesting feature of the 53 nm thick film. There was a network of characteristic cracks present on their surface (Figure 2, right panels). They extended from the ray of one snowflake to the ray of another snowflake along the characteristic directions {1–10}, occasionally changing direction to another one. Most likely, they were the result of the plastic relaxation of the film. The longer the time of electronic exposure, the greater the density of these cracks. On the surface of the film with a smaller thickness of 29 nm, such cracks were practically absent. Two reasons could be responsible for the crack formation: the strain in the film due to a difference in the CaSi_2_ and CaF_2_ lattice constants and the overpressure of free fluorine produced in the film volume during electron irradiation. The longer the time of electron exposure, the larger the CaSi_2_ structures incorporated in the CaF_2_ film. Figure 2d demonstrates the surface of a 53 nm thick sample after a 4 min electron irradiation. In this case, the CaSi_2_ structures became three-dimensional objects embedded in CaF_2_ film. They squeezed CaF_2_ between them and caused the appearance of cracks. The amount of free fluorine stored in the film also increased with exposure time, which could have also led to the film cracking.

**Figure 4:**
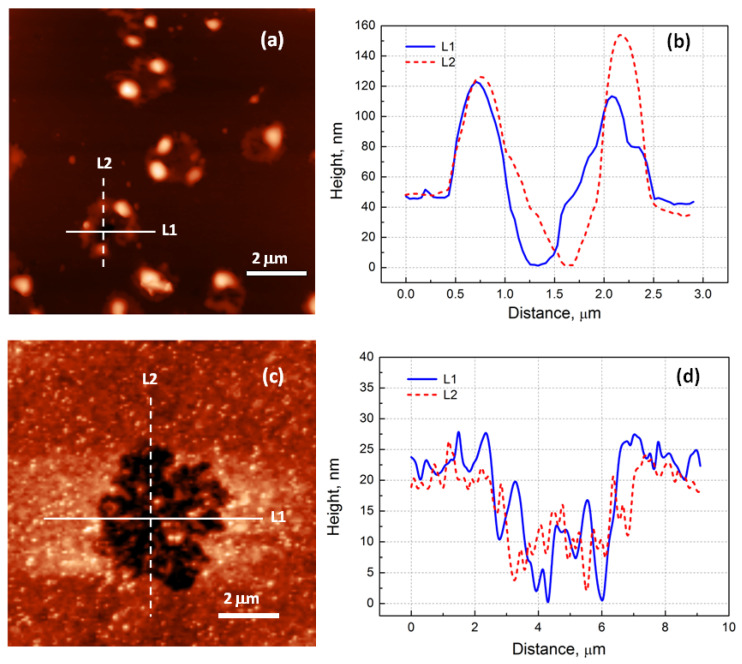
AFM data obtained after a 1 min electron irradiation: (**a**)—29 nm thick film; (**c**)—53 nm thick film. Profiles of surface relief along L1 and L2 directions: (**b**)—29 nm thick film; (**d**)—53 nm thick film.

The AFM study helped us to understand how snowflakes formed. Figure 4 shows the morphology of the surface after a 1 min electron irradiation for both structures. We could see that craters formed on the film’s surface. The shape and size of the craters were different and consistent with the SEM results. For the first structure, the depth of the crater was close to the thickness of the epitaxial film. The crater walls were formed with broken pieces of the epitaxial film. The total height of the walls from the bottom of the crater was, on average, 130 nm. For the second structure, the craters were flatter, and their depth was approximately two times smaller than the film’s thickness. Such a picture suggested the following interpretation: CaF_2_ decomposed into calcium and fluorine in the whole volume of the film, since the electron beam at the used energies penetrated the entire depth of the film. Interstitial fluorine atoms could move rapidly along the anion close-packed direction via a replacement sequence [29,30]. Parts of the fluorine atoms came to the surface and were desorbed [31,32]. Fluorine vacancies surrounded by Ca (*F* centers) diffused to the surface [33,34] and could meet on their way the embedded Si layers. Ca atoms could bind to the Si atoms, and two-dimensional CaSi_2_ islands (or sections of two-dimensional CaSi_2_ layers) could form. The remaining parts of the fluorine atoms collected in certain places near the defects, which could be twin boundaries or stacking faults in the CaF_2_ film. The highest defect density was observed at the CaF_2_/Si heterointerface, so parts of the fluorine atoms accumulated near the bottom of the film, forming fluorine bubbles (see the detailed study of bubble formation in Ref. [31]). When the critical overpressure was reached, there was an explosion and the formation of a crater. In the following stages, due to the diffusion and electron irradiation, the crater walls flattened, and the material of the walls was distributed around the crater. The crater itself could be filled with some material such as a calcium-silicon compound (see EDX data, Figure 3). As long as the crater was not filled, intense silicon diffusion from the substrate could occur along the crater walls. All this led to the formation of structures resembling snowflakes, which can be seen in Figure 2 and Figure 3. The difference in the size and density of calcium silicide structures observed for samples with different thicknesses could be explained by the different amounts of fluorine produced in the volume of CaF_2_ films. The amount of fluorine per “nucleation” center, where calcium silicide structures were formed, was several times greater for a 53 nm thick film, so the explosions occurred at an earlier stage, leading to a larger size of calcium silicide crystallites. The density of explosions was lower for the thicker film, because only the larger fluorine bubbles burst through.

**Figure 5:**
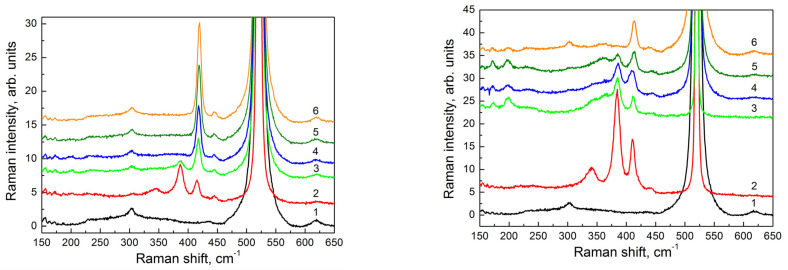
Raman spectra obtained for structures with different thicknesses of epitaxial films; left panel: 29 nm; right panel: 53 nm. Spectrum (1) corresponds to a Si(111) substrate, spectrum (2) corresponds to a film grown with simultaneous electron irradiation during deposition of CaF_2_. Other spectra correspond to regions of the films formed with postgrowth electron irradiation with different electron exposure times: (3) 10 min; (4) 4 min; (5) 2 min; (6) 1 min.

According to the Raman data, a narrow intensive peak at ≈ 419 cm^−1^ (Figure 5, left panel, spectrum six) was observed for a 29 nm thick film at the minimum irradiation time (1 min). This peak corresponded to vibrations of Si atoms in the plane of the calcium-intercalated two-dimensional Si layer [35] that supported the assumption about the formation of the CaSi_2_ two-dimensional layer (or its regions). With an increasing irradiation time, the intensity of this peak decreased, and its position slightly shifted to the lower frequencies. At a 10 min irradiation, the peak intensity fell by a factor of three, and the peak position was ≈ 417.5 cm^−1^ (Figure 5, left panel, spectrum three). At the same time, an additional peak appeared at ≈ 386 cm^−1^.

An analogous peak with the same position was observed in the Raman spectrum from the CaSi_2_ film obtained with electron beam irradiation during the growth of CaF_2_ layers (Figure 5, left panel, spectrum two). This peak corresponded to the vibrations of Si atoms in the direction perpendicular to the plane of a two-dimensional calcium-intercalated Si layer [35]. These changes indicated the appearance of three-dimensional CaSi_2_ structures in the studied films at sufficiently long irradiation times. For a 53 nm thick film, a peak corresponding to vibrations of Si atoms in the direction perpendicular to the plane of the two-dimensional calcium-intercalated Si layer was already observed in the Raman spectrum of the sample with a 2 min electron irradiation (Figure 5, right panel, spectrum five). The peak amplitude increased with the increasing irradiation time, indicating the appearance of three-dimensional structures at earlier stages of irradiation as compared to a 29 nm thick film.

**Figure 6:**
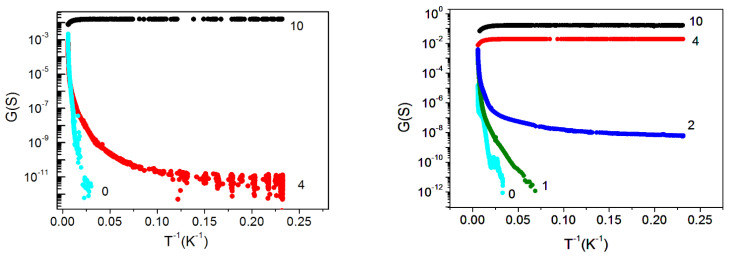
Temperature dependence of conductivity G for structures with different thicknesses of epitaxial films. **Left** panel:29 nm thick film; **right** panel:53 nm thick film. The numbers next to the experimental curves correspond to the electron exposure time measured in minutes.

The conductance measurements of the films under study (Figure 6) were in good agreement with the SEM and EDX data. The conductance increased with the electron exposure time, with the temperature dependence of the conductivity changing from the semiconductor to the metal one. For the same electron irradiation time, the conductance of the thicker sample was always larger than that of the thinner one. Correspondingly, a 53 nm thick sample already exhibited a metallic behavior after a 4-min irradiation, while a 29 nm thick sample behaved like a semiconductor at this irradiation time.

To understand how the conductivity of the samples with short times of electron irradiation (2 min for a 53 nm thick film and 4 min for a 29 nm thick film) was realized, we needed to take into account that the samples were nonuniform—most likely, they contained metallic clusters of different sizes and densities. As can be seen from the SEM and EDX data (Figure 2 and Figure 3), the distance between visible CaSi_2_ crystallites at a short irradiation time was quite large for a measurable conductivity. Moreover, as-grown CaF_2_ film without electron irradiation behaved like an insulator; thus, to explain the observed experimental data, one had to assume that small clusters, not distinguishable with SEM and EDX, were formed between large CaSi_2_ crystallites. This assumption is supported by a number of works [32,36] showing that electron irradiation causes the near-surface region of CaF_2_ to become conductive. The results [33] demonstrated that electron irradiation stimulates the formation of *F* centers, the diffusion of which to the surface leads to its enrichment with calcium. In the near-surface region, metallic colloidal pieces were formed, which provided conductivity. Similar results were obtained after the ultraviolet irradiation of CaF_2_ film on Si(111) [37]. We supposed that, in our samples with short irradiation times, the conductivity between large CaSi_2_ crystallites was carried out through transitions between these small metallic clusters. This feature seemed to determine the conductivity behavior with decreasing temperatures in these samples (Figure 6). With an increasing irradiation time, the size and number of CaSi_2_ crystallites increased, leading to their convergence and overlapping, respectively; film conductivity increased too, going to a metallic one.

**Figure 7:**
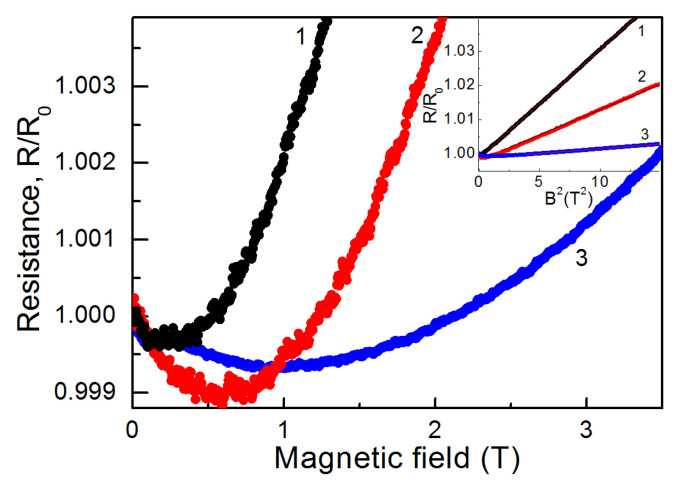
Magnetoresistance of films formed with postgrowth electron irradiation with different times of electron exposure. Curves 1 and 2 are related to the film with a thickness of 53 nm after electron irradiation for 10 min and 4 min, respectively. Curve 3 corresponds to the film with a thickness of 29 nm after electron irradiation for 10 min.

Figure 7 demonstrates the magnetoresistance (MR) of the 53 nm thick samples after 4 and 10 min of electron irradiation and the MR of the 29 nm thick sample after 10 min of electron irradiation. One could see that the behavior of MR was qualitatively similar for these samples. In low magnetic fields, we observed a negative MR that crossed over to a positive one as magnetic field **B** increased. The transition from the negative to the positive MR depended on the sample thickness and the electron irradiation time. The greater the conductivity of the sample, the faster the change in the MR sign. For the less conductive samples (29 nm thick samples), this took place in the stronger fields. A negative MR is usually associated with the suppression of weak localization in the magnetic field [38]. A positive MR in metallic samples is due to the Lorentz deflection of carriers [39].

According to the Kohler rule [40], in this case, *R*(*B*)/*R*(*B* = 0) ∼ 1 + (*μB*)^2^. From the slope of *R/R_0_(B^2^)* dependence (inset to Figure 7), we could estimate the carrier mobility *μ*. For a 53 nm thick film, *μ* was found to be ~560 cm^2^ V^−1^ s^−1^ for the sample with a 10 min electron irradiation and ~380 cm^2^ V^−1^ s^−1^ for the sample with a 4 min electron irradiation, correspondingly. For a 29 nm thick sample irradiated for 10 min, *μ*~ 165 cm^2^ V^−1^ s^−1^.

The obtained results allowed us to reconstruct the scheme of the CaSi_2_ formation under the electron irradiation of already-grown CaF_2_ films. The electron irradiation led to a strong excitation of the film atomic system. The bonds between calcium and fluorine atoms broke. One part of the fluorine atoms reached the surface and were desorbed. The other part accumulated near defects and then escaped from the film during the explosions. Fluorine vacancies surrounded by Ca (*F* centers) diffused to the surface [33] and could meet on their way the embedded Si layers. At this stage calcium atoms, bound with silicon atoms from these layers and formed the two-dimensional CaSi_2_ regions. In the next stages, after the explosions, the number of Si atoms increased due to diffusion from the substrate along the crater walls, and the three-dimensional CaSi_2_ crystallites began to form. With an increasing irradiation time, the surface density of polycrystalline structures became larger, and their size increased, finally resulting in the formation of a continuous metallic conducting layer.

## 4. Conclusions

The obtained results demonstrated that the postgrowth electron irradiation of CaF_2_ films with embedded thin Si layers could be used to produce CaSi_2_ structures, including two-dimensional ones. By adjusting the energy of electrons, the time of electron exposure, the thickness of the CaF_2_ film and the temperature of the substrate, it was possible to find the optimal conditions for the formation of two-dimensional CaSi_2_ structures under electron beam irradiation. The key point was to prevent the formation of fluorine bubbles, which provided the appearance of three-dimensional CaSi_2_ structures.

## Data Availability

Data are contained within the article.
